# Development and validation of a model for predicting upstage in minimally invasive lung adenocarcinoma in Chinese people

**DOI:** 10.1186/s12957-024-03414-5

**Published:** 2024-05-22

**Authors:** Yida Liao, Zhixin Li, Linhong Song, Yang Xue, Xiangru Chen, Gang Feng

**Affiliations:** 1 Department of Thoracic Surgery, School of Medicine, Sichuan Provincial People’s Hospital, University of Electronic Science and Technology of China, Chengdu, China; 2 Department of Thoracic Surgery, Shanghai Pulmonary Hospital, School of Medicine, Tongji University, Shanghai, 200433 P.R. China; 3 Department of Pathology, School of Medicine, Sichuan Provincial People’s Hospital, University of Electronic Science and Technology of China, Chengdu, China

**Keywords:** Non-small cell lung cancer, Frozen section, Microinvasive adenocarcinoma, Surgery, Pathological upstages

## Abstract

**Background:**

Sublobar resection for ground-glass opacity became a recommend surgery choice supported by the JCOG0804/JCOG0802/JCOG1211 results. Sublobar resection includes segmentectomy and wedge resection, wedge resection is suitable for non-invasive lesions, but in clinical practice, when pathologists are uncertain about the intraoperative frozen diagnosis of invasive lesions, difficulty in choosing the appropriate operation occurs. The purpose of this study was to analyze how to select invasive lesions with clinic-pathological characters.

**Methods:**

A retrospective study was conducted on 134 cases of pulmonary nodules diagnosed with minimally invasive adenocarcinoma by intraoperative freezing examination. The patients were divided into two groups according to intraoperative frozen results: the minimally invasive adenocarcinoma group and the at least minimally invasive adenocarcinoma group. A variety of clinical features were collected. Chi-square tests and multiple regression logistic analysis were used to screen out independent risk factors related to pathological upstage, and then ROC curves were established. In addition, an independent validation set included 1164 cases was collected.

**Results:**

Independent risk factors related to pathological upstage were CT value, maximum tumor diameter, and frozen result of AL-MIA. The AUC of diagnostic mode was 71.1% [95%CI: 60.8-81.3%]. The independent validation included 1164 patients, 417 (35.8%) patients had paraffin-based pathology of invasive adenocarcinoma. The AUC of diagnostic mode was 75.7% [95%CI: 72.9-78.4%].

**Conclusions:**

The intraoperative frozen diagnosis was AL-MIA, maximum tumor diameter larger than 15 mm and CT value is more than − 450Hu, highly suggesting that the lung GGO was invasive adenocarcinoma which represent a higher risk to recurrence. For these patients, sublobectomy would be insufficient, lobectomy or complementary treatment is encouraged.

## Background

The 2015 edition of the WHO classification of lung adenocarcinoma basically adopts the standard of the classification of lung adenocarcinoma proposed by the International Lung Cancer Research Association (IASLC), American Thoracic Society (ATS) and European Respiratory Society (ERS) in 2011 [1, 2]. The application of this classification has led to significant changes in the diagnosis and treatment of lung adenocarcinoma, especially in the diagnosis and treatment of early lung adenocarcinoma. This edition classifies lung adenocarcinoma into the following types: pre-invasive lesions (atypical adenomatous hyperplasia, adenocarcinoma in situ), MIA (Micro-invasive adenocarcinoma), invasive adenocarcinoma, and variants of invasive adenocarcinoma. The above classification provides a detailed histological description of each type of pathological diagnosis, but there are still great challenges in clinical practice. According to the actual clinical situation, the Chinese Society of Pathology (CSP) classified nonmucinous MIA into the following categories [3]: (1) conforming to MIA; (2) having a high probability of MIA; and (3) at least MIA, with no exception of infiltrating adenocarcinoma (AL-MIA). The diagnosis of AL-MIA is defined as follows: the tumor cells mainly grow attached in a wall in frozen sections, some areas exhibit clear interstitial infiltration, but the infiltration range is uncertain, and further paraffin section diagnosis is needed.

The operation choice in early-stage lung cancer can be divided into lobectomy and sublobectomy [3], as the JCOG0804/COG0802/ JCOG1211 [4, 5] results released, sublobectomy (segmentectomy and wedge resection) become a standard treatment for GGO. However, the conditions for the use of wedge resection are highly controversial. Evidence showed in stage I patients [6, 7], segmentectomy showed comparable survival outcomes and recurrence patterns to lobectomy and was superior to wedge resection. Wedge resection is non-anatomical procedure, which contribute to elevated local recurrence rates. In order to select invasive lesions in uncertain frozen results patients, we conducted a retrospective study.

## Methods

### Patients and samples

A retrospective study was conducted on 134 cases of pulmonary GGN diagnosed with MIA by intraoperative frozen section in our hospital in 2019. The informed consent was obtained for experimentation before surgery. The pathological results of postoperative paraffin sections were collected and divided into two groups according to frozen section: the minimally invasive group and the AL-MIA group. A variety of clinical features with potential value in the diagnosis of malignant GGNs were collected according to literature reports [6, 7]: maximum diameter, regular shape, lobulation, spiculation, pleural retraction, vacuoles, air bronchogram, vascular Convergence, clear border, solid ingredients (Mediastinal window setting, any percentage), CT value, etc.

An independent validation sets included 300 Mia and 864 AL-MIA cases from Shanghai Pulmonary Hospital was collected, the operation date ranged from 2019 to 2022.

### Statistical analysis

SPSS 22.0 was used for statistical analysis, and *P* < 0.05 indicated a significant difference. The chi-square test was used to calculate the difference in different parameters between the two groups. The single factor chi-square test was used to calculate the correlation between different parameters. A multiple regression logistic method was used to screen out the independent risk factors related to the upgrading of pathological results. Based on the results of multiple regression, the ROC curve was built. In addition, 1164 cases of pulmonary nodules diagnosed as AL-MIA and MIA by intraoperative freezing examination was collected as a validation set to verify the accuracy of the regression equation. Written informed consent for clinical research from the patient was obtained before surgery. All the frozen sections and final pathology was reviewed by same pathologist.

## Results

### Patients characteristics

The clinical characteristics of the 134 patients with GGO included in this study are listed in Table 1. The number of tumors classified as MIA and AL-MIA are 67 and 67 respectively. There are significant differences (*P* < 0.05) in the following clinicopathological features between the MIA group and AL-MIA group: edge smoothing, pleural retraction, regular shape, spiculation, solid components, and pathological upstage.

**Table 1:** Clinical characteristic of different groups

Clinical features	MIA	AL-MIA	χ^2^(variance)	*P* values
Gender (Male/Female)	18/49	20/47	0.15	0.70
Age 40+ (Y/N)	55/12	60/7	1.53	0.22
Smoking history (Y/N)	10/57	10/57	0.00	1.00
Family cancer history (Y/N)	13/54	13/53*	0.00	0.97
Clear border (Y/N)	16/51	7/60	4.25	0.04
Pleural retraction (Y/N)	17/50	31/36	6.36	0.01
Regular shape (Y/N)	32/35	19/48	5.35	0.02
Lobulation (Y/N)	30/37	30/37	0.00	1.00
Spiculaion (Y/N)	26/41	38/29	4.30	0.04
Vacuoles (Y/N)	50/17	46/21	0.59	0.44
Air bronchogram (Y/N)	58/9	63/4	2.13	0.14
Vascular Convergence (Y/N)	48/19	58/9	4.51	0.03
Solid ingredients (Y/N)	9/58	22/45	7.09	0.01
Maximum diameter^★^(Mean ± SD)	0.96 ± 0.51	1.12 ± 0.47	13.96	0.00
CT value (Mean ± SD)^▲^	-482.15 ± 171.54	-449.51 ± 208.33	0.15	0.70
Pathological upstage (Y/N)	7/60	30/37	19.75	0.00

Y/N: Yes/No. Pathological upstage: non-invasive lesions in frozen section and invasive lesions in paraffin section

### Associations between pathological upstages and clinical factors

Univariate analysis revealed that the risk factors associated with pathological upstages were pleural retraction, regular shape, lobulation, spiculation, solid components, maximum diameter, CT value, and AL-MIA frozen sections [Table 2].

**Table 2:** Single factor Chi-square test with pathological upstage

Predictors	χ2	*P* values	
Age40+(Y/N)	0.48	0.49	
Smoking history (Y/N)	0.07	0.80	
Family history(Y/N)	0.22	0.63	
Clear border (Y/N)	0.48	0.49	
Pleural retraction (Y/N)	12.42	0.00	
Regular shape (Y/N)	5.86	0.02	
Lobulation, (Y/N)	4.46	0.04	
Spiculaion (Y/N)	8.04	0.01	
Vacuoles (Y/N)	1.14	0.29	
Air bronchogram (Y/N)	0.15	0.70	
Vascular Convergence (Y/N)	0.68	0.41	
Solid ingredients (Y/N)	22.89	0.00	
Maximum diameter	26.43	0.00	
CT value	20.29	0.00	
Frozen results (Mia/AL-Mia)	19.75	0.00	

**Fig. 1:**
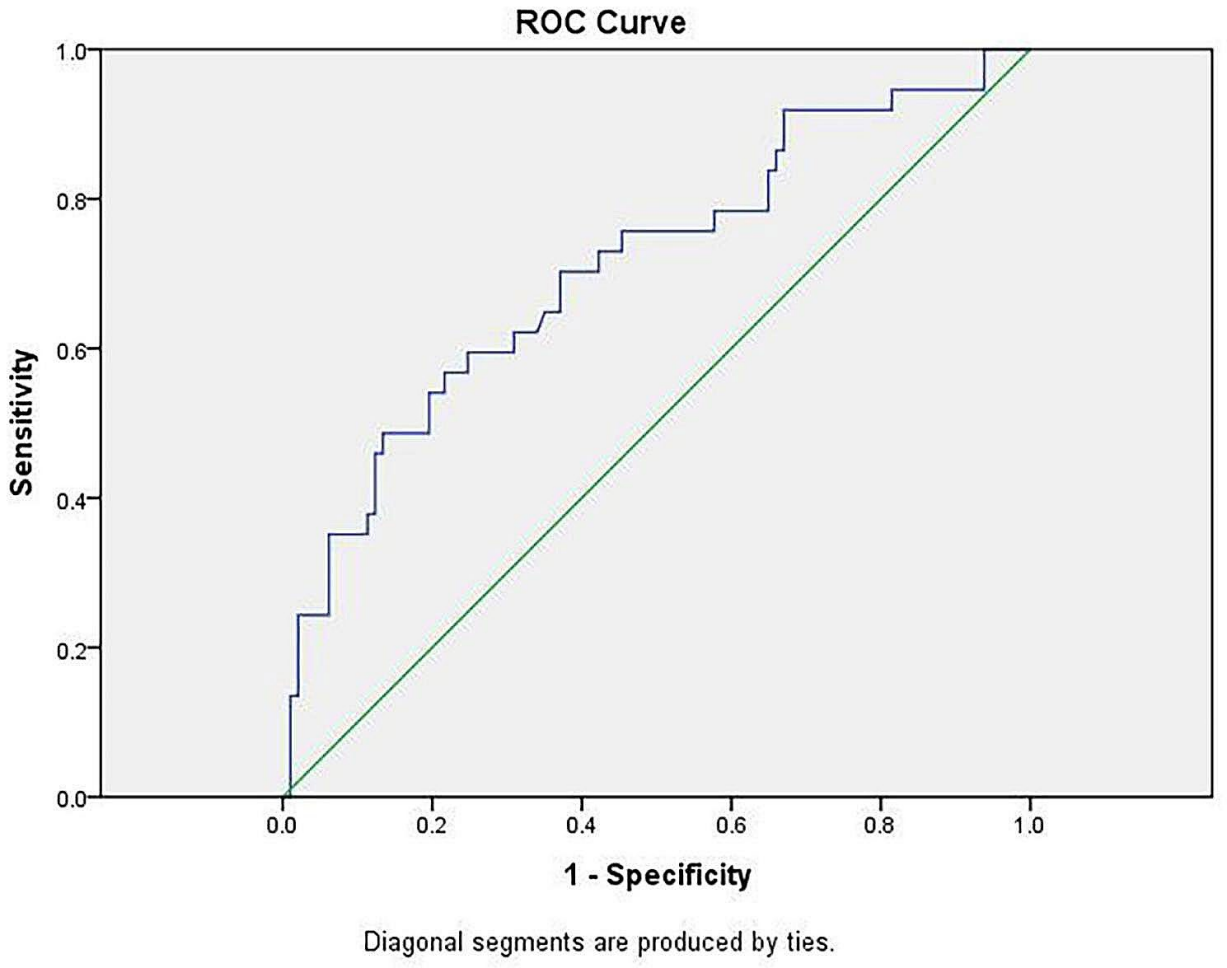
ROC curve for predicting pathological upstage in MIA patients

Multivariate regression selected independent risk factors related to the pathological upstage: CT value, maximum diameter, AL-MIA frozen sections [Table 3], and the prediction equation was as follows: pathological upstage = 0.005×CT value + 1.60×maximum diameter − 1.94× AL-MIA frozen sections-0.04, and the area under ROC curve was 71.1%(SE = 0.05, *P* = 0.00, 95% CI [0.61, 0.81]) (Fig. 1).

**Table 3:** Multivariate analysis with pathological upstage

Variables	HR (95% CI)	*P* values
CT value	1.00(1.00-1.01)	0.02
Maximum diameter,	6.99(2.06–23.71)	0.00
Frozen results (Mia/AL-Mia)	0.16(0.05–0.50)	0.00

The logistic multiple regression model is used as the diagnostic standard to predict whether the frozen sections of MIA are upstaged in final result. The area under the curve:0.71(SE = 0.05).

### Independent validation

**Fig. 2:**
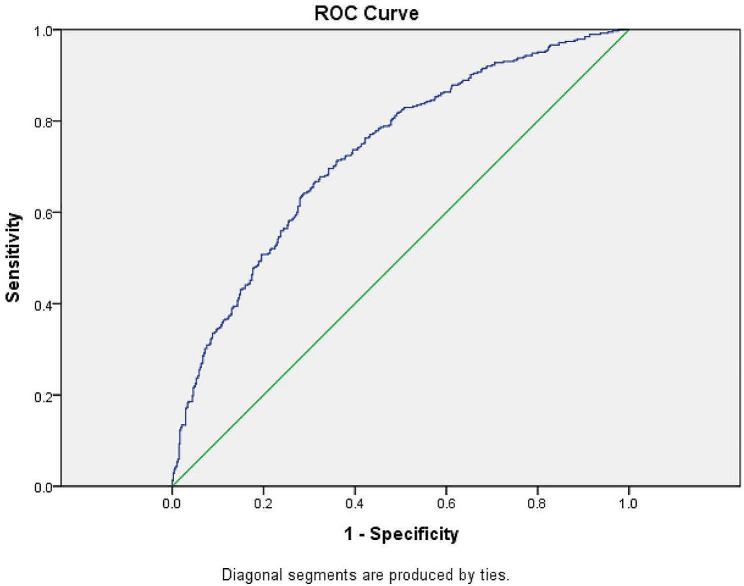
ROC curve for predicting pathological upstage in validation set

The independent validation set included 864 AL-MIA patients and 300 MIA patients, the pathological up-grade rate were 388/864(45%) and 29/300(9%) respectively. Multivariate regression selected independent risk factors related to the pathological upstage: CT value, maximum diameter, frozen results [Table 4], and the prediction equation was as follows: pathological upstage = -1.10 ×CT value − 1.22 ×maximum diameter − 1.65×frozen results + 0.67, The AUC of diagnostic mode was 75.7% [95%CI: 72.9-78.4%][Fig. 2].

**Table 4:** Selected clinical characteristic of validation set

Clinical features	Pathological upstage (*N*)	Pathological upstage (Y)	χ^2^ /variance	*P* values
Maximum diameter^★^(Mean ± SD)	11.76 ± 4.54	15.33 ± 5.61	20.21	0.00
CT value (Mean ± SD)^▲^	-540.37 ± 129.85	-456.92 ± 152.67	13.56	0.00
Frozen results (Mia/AL-Mia)	271/476	29/388	120.29	0.00

t-test for Equality of Means

The logistic multiple regression model is used as the diagnostic standard to predict pathological upstage with selected variables (Maximum diameter, CT value and Frozen result). The area under the curve:0.76(SE = 0.01).

## Discussion

Surgery recommendations for non-invasive and invasive adenocarcinoma are different, patients who undergo lobectomy have superior overall and cancer-specific survival rates, regardless of tumor size [8–13]. In clinical practice, intraoperative frozen sections will determine the specific mode of operation, thus the accuracy of intraoperative frozen sections will directly influence the patient’s prognosis [14], however, pathological upstages occur for various reasons [15], research findings found that complementary treatment is encouraged in AIS/MIA upstaged to invasive adenocarcinoma by final pathology after sublobectomy [16].

In this study, intraoperative frozen result was an independent risk factor for the pathological upstage of MIA after surgery, and other independent factors included maximum diameter and CT value. The prediction model established by these three risk factors achieved a reliable accuracy in training set and independent validation set respectively.

The frozen and paraffin diagnosis of adenocarcinoma is not always the same [17, 18], the overall concordance rates between FS and FP were 79.1% (κ = 0.650) and 89.6% (κ = 0.729) with substantial agreement in retrospective and prospective cohorts, respectively [19], The reasons mainly include the followings: (1) Ground glass nodules include some chronic inflammatory lesions and fibrosis lesions, which contain alveolar cell hyperplasia, and these disturbed frozen pathological diagnoses. (2) The 2015 edition of the WHO classification described the histological characteristics of paraffin sections in detail. However, the ideal example cannot guide complex pathological practice, which causes substantial mental pressure on pathologists. (3) It is also very difficult to distinguish MIA from a lepidic predominant invasive ADC using frozen section. On frozen section slides, alveolar spaces are frequently collapsed, which can cause difficulty in evaluating invasion [20].(4) It is difficult to identify high risk histologic features such as micropapillary subtype and tumor spread through air space (STAS) on frozen Sect. [20].

In 2015, the Sloan Cancer Center in New York showed that the accordant rate between frozen and paraffin diagnosis was only 68% in lung cancer, while the average accuracy rate was 64% (54%~74%) in frozen diagnosis of invasive degree in early stage lung adenocarcinoma [17]. The correct judgment of non-invasive and invasive lesions is of great significance for surgical choice because non-invasive lesions rarely metastasize after surgery, whereas invasive lesions metastasize earlier. For the above reasons, pathologists cannot distinctively distinguish these two conditions, which will result in difficulties in choosing the surgery type.

**Fig. 3:**
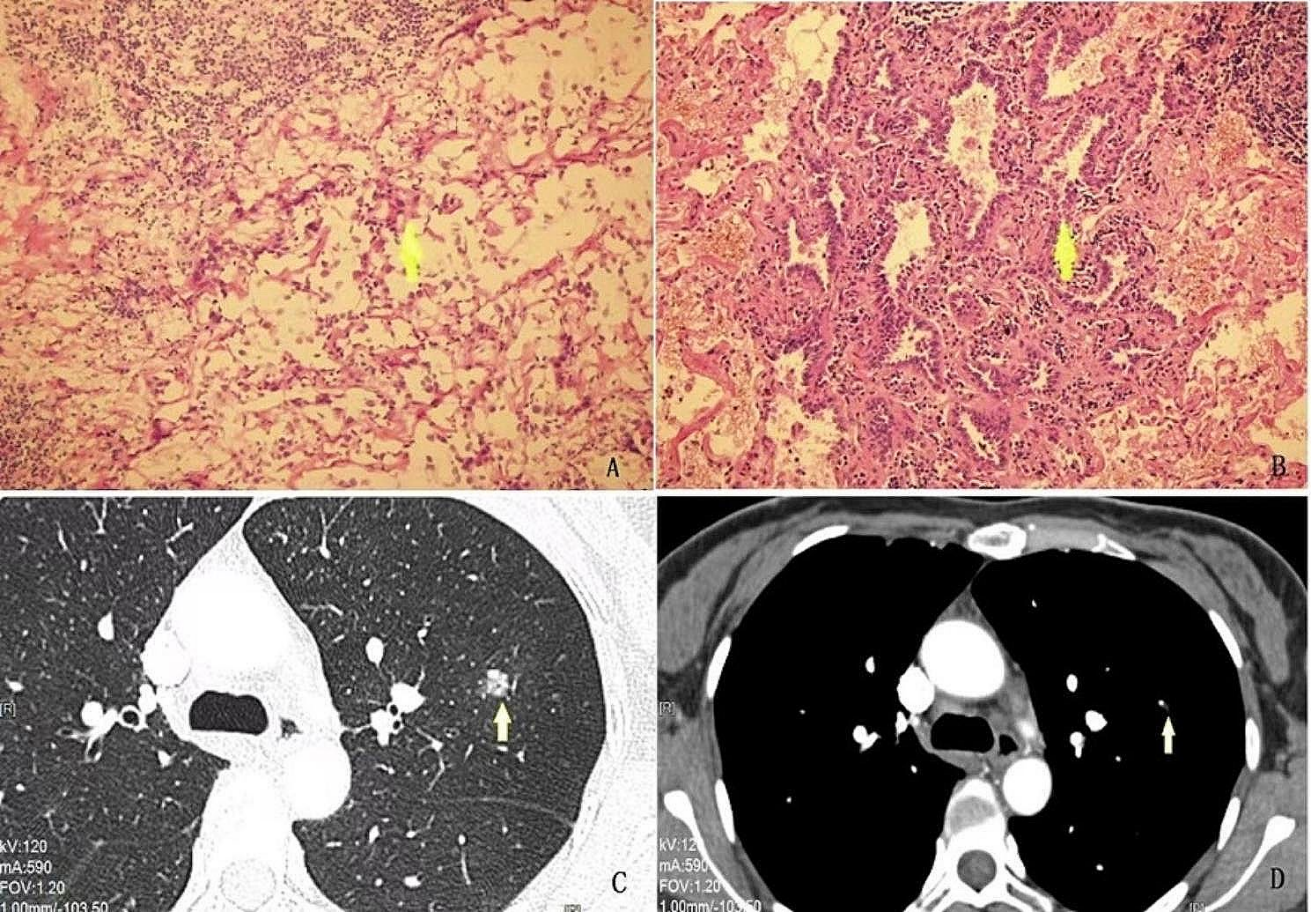
Pathological and CT image characteristic of pathological upstage in a patient. **A**: AL-MIA in frozen section. **B**: Invasive adenocarcinoma in paraffin section. **C**: Lung window in CT. **D**: Mediastinal window in CT

When pathologists diagnose MIA in frozen sections, some cases may represent typical invasive adenocarcinoma performance, but some cases are not. The Chinese Pathological Association divides the non-mucous type of MIA in frozen diagnosis into three kinds: (1) accompanying MIA; (2) the possibility of MIA is high; and (3) AL-MIA. The definition of AL-MIA is as follow [3]: in frozen sections, tumor cells mainly exhibit wall-like growth, and there is clear stromal infiltration; however, it was impossible to assess whether the maximum diameter of the infiltration area was > 0.5 cm. At this time, it could be diagnosed as AL-MIA, and paraffin section diagnosis should be further clarified. This diagnostic term is mainly suitable for cases in which tumor cells can be clearly seen infiltrating into the lung stroma under frozen sections. However, due to the limitations of the frozen section, the infiltration scope cannot be completely determined, and further determination should be made after all samples are taken. Most of these cases are due to the poor quality of frozen sections, which makes it difficult to accurately assess the size of the invasive lesion (Fig. 3).

In some cases, the invasive lesions were relatively large, but neither CT imaging nor frozen sections could completely determine whether the diameter exceeded the standard of 0.5 cm. In a small number of cases, multipoint minimally invasive lesions exist in the tumor, so the invasive range should be determined by multiplying the sum of the percentage of each invasive focus in the total tumor volume by the maximum diameter of the tumor. In addition, some well-differentiated acinar adenocarcinomas can grow like walls, so frozen sections are easily confused with adenocarcinoma in situ with fibrosis.

All of the above will be completed on paraffin sections after all tumor samples are collected, if pathological upstages occur, surgeons should timely communicate with the patient’s family members, because tumor invasion (invasive adenocarcinoma [IAD] vs. adenocarcinoma in situ [AIS]/minimally invasive adenocarcinoma [MIA]) was the only independent predictor for 5-year recurrence free survival [21], a complementary surgery might be recommended.

## Conclusions

With the finding of this research, pathologists and thoracic surgeons can use this predicting model to avoid underestimation and potentially insufficient resection. The intraoperative frozen diagnosis was AL-MIA, maximum tumor diameter larger than 15 mm and CT value is more than − 450Hu, highly suggesting that the lung GGO was invasive adenocarcinoma which represent a higher risk to recurrence. For these patients, sublobectomy would be insufficient, lobectomy or complementary treatment is encouraged.

## Data Availability

No datasets were generated or analysed during the current study.

## Abbreviations

MIA: Micro-invasive adenocarcinoma
AL-MIA: At Least Minimally Invasive Adenocarcinoma
ROC: Receiver Operating Characteristic Curve
AUC: Area under curve
GGN: Ground Glass Nodule
CT: Computed Tomography
SE: Standard Error
HR: Harzard Ratio
CI: Confidence Intervals
Y/N: Yes/No
